# A Multifunctional Light-Driven Swimming Soft Robot for Various Application Scenarios

**DOI:** 10.3390/ijms23179609

**Published:** 2022-08-25

**Authors:** Zhen Wang, Dongni Shi, Xiaowen Wang, Yibao Chen, Zheng Yuan, Yan Li, Zhixing Ge, Wenguang Yang

**Affiliations:** 1 School of Electromechanical and Automotive Engineering, Yantai University, Yantai 264005, China; 2 State Key Laboratory of Robotics, Shenyang Institute of Automation, Chinese Academy of Sciences, Shenyang 110016, China

**Keywords:** bionic swimming soft robot, micromanipulator, light-driven, flexible actuator, light-responsive hydrogel, DMD, poly-N-isopropylacrylamide (PNIPAM)

## Abstract

The locomotor behavior of creatures in nature can bring a lot of inspiration for the fabrication of soft actuators. In this paper, we fabricated a bionic light-driven swimming soft robot that can perform grasping of tiny objects and achieve the task of object transfer. By adding carbon nanotubes (CNTs), the temperature-sensitive hydrogels can be endowed with light-responsive properties. The fabricated composite hydrogel structure can control the contraction and expansion of volume by light, which is similar to the contraction and diastole behavior of muscles. The oscillation of the fish tail and the grasping action of the normally closed micromanipulator can be achieved by the control of the irradiation of the xenon light source. The bending of the bionic arm can be controlled by the irradiation of a near-infrared (NIR) laser, which transforms the spatial position and posture of the micromanipulator. The proposed scheme is feasible for miniaturized fabrication and application of flexible actuators. This work provides some important insights for the study of light-driven microrobots and light-driven flexible actuators.

## 1. Introduction

Traditional mechanical devices are mainly powered by electric motors or engines, which can provide various driving forces to mechanical devices or robots through various sophisticated forms of mechanical transmission. In the production of microrobots, various physical and chemical fields are needed to accomplish microscale motion and deformation [1]. By responding to various stimuli, such as temperature [2], light [3], pH [4], humidity [5], magnetic fields [6,7,8,9], etc., the robot can be provided with energy for movement, and certain motions can be accomplished. Soft actuators have received increasing attention in the study of bionic robots [10,11,12]. In nature, many organisms rely on soft tissues to achieve flexible drives to perform complex and precise movements. Studying biological brake systems and learning from nature may inspire the generation of new mechanisms and structures for the operation of flexible brakes.

With the help of the development of functional materials and the rational design of mechanical structures, it is possible to imitate the flexible movement behavior of some living organisms in nature [11,13,14]. Macroscopic aspects are commonly studied with pneumatic manipulators [10]. Microscopic aspects are also an effective form of flexible actuation by directly incorporating living biological components, such as cardiomyocytes, in abiotic structures [15]. Bingzhe Xu et al. created a bio-hybrid planar swimming robot by growing a cardiac microstructure on a bio-affinity material and using it as a tail fin to mimic the swimming of a whale [16]. Polymer hydrogels that can respond to external stimuli can also be used as actuators in applications such as microrobotics [17,18,19]. One of the most widely used temperature-responsive hydrogels is PNIPAM hydrogel, which has a low critical solution temperature (LCST) of approximately 32 °C [20,21,22,23]. The N-isopropylacrylamide molecule has both a hydrophilic amide group and a hydrophobic isopropyl group. When the temperature is lower than LCST, the hydrogel exhibits hydrophilicity due to increased intermolecular forces between polymer chains and water molecules, and the hydrogel absorbs water and swells. When the temperature is higher than LCST, PNIPAM shifts from hydrophilic state to hydrophobic state due to the weakening of the interaction between polymer and water molecules, and the hydrogel expels water and shrinks in volume. Temperature can affect the contraction and expansion of the volume of temperature-sensitive hydrogels, and its characteristics are similar to the contraction and diastole of muscle tissue. Therefore, temperature-sensitive hydrogels have good application prospects for flexible brakes. However, direct control of the deformation behavior of temperature-sensitive hydrogels by temperature makes it difficult to achieve local stimulation of the precise site. Combining photothermal conversion materials with temperature-sensitive hydrogels can greatly expand the control scheme of temperature-sensitive hydrogel deformation. Light is able to achieve large overall irradiation areas or can be focused to tiny areas of tens of nanometers. NIR light-driven microrobots can be used inside the body for drug transport and release [24]. For robots, the use of light energy to drive the motion of the robot has good prospects for application.

In this paper, we combined CNTs with PNIPAM hydrogels to fabricate a photo-responsive hydrogel material. CNTs have good full-spectrum light absorption ability and relatively high photo-thermal conversion ability, which have been more used in photo-deformable materials [25,26,27,28,29]. By converting light to heat, the volume change in a temperature-sensitive hydrogel is indirectly driven by the change in temperature to produce water drainage and water absorption. The hydrogel structures are cured by a prepolymer solution through a digital light processing-based light-curing 3D printing technique. This approach has the advantage of rapid prototyping of complex structures and high fabrication accuracy in the fabrication of microrobots. Using PNIPAM/CNT composite hydrogel material as a flexible actuator, we fabricated the light-controlled reciprocating oscillating fish tail. The oscillation of the fish tail is used to provide the power for the fish to move forward. Drawing on the idea of 4D printing, a light-responsive open and normally closed micromechanism hand was fabricated. By adding a bionic arm and a micromanipulator, the swimming soft robot can be made to have the ability to grasp objects and transfer them. The above-mentioned scheme of making a bionic microfish that can perform object grasping and swimming provides a new driving scheme for the research of underwater microrobots.

## 2. Results and Discussion

### 2.1. Fabrication of Light-Responsive Microstructures

**Figure 1:**
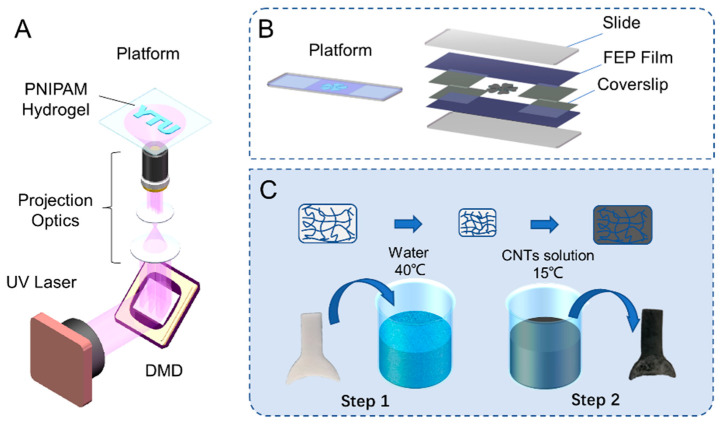
(**A**) Schematic diagram of the light-curing system based on the digital micromirror device. (**B**) Customized light-curing platform for hydrogel microstructures. The hydrogel structure is cured between two FEP films to complete. (**C**) Steps of PNIPAM hydrogel impregnation of CNTs.

The temperature-sensitive hydrogel PNIPAM exhibits drastic volume changes above and below 32 °C. The addition of CNTs to the temperature-sensitive hydrogel enables the PNIPAM/CNT composite hydrogel to have light-driven volume change properties through the good photothermal conversion ability of CNTs. Four-dimensional printing, based on three-dimensional printing, allows the printed structure to be transformed from one shape to another through various physical or chemical stimuli after the structure is made. Light-driven functional morphing materials are also a form of 4D printing. Complex forms of deformation or driven forms require systems that are fabricated with the ability to produce complex structures. The light-curing technology based on digital micromirror device (DMD) has higher printing accuracy and printing speed compared to the ordinary nozzle form of 3D printing technology. By projecting the design photomask UV pattern, it can cure the whole layer of the structure at the same time. For the fabrication of the hydrogel structure, we use a light-curing system as shown in Figure 1A. The curing system mainly includes DMD, UV light source, projection light path system, and curing platform. As shown in Figure 1B, the prepolymer solution of the hydrogel is placed between two fluorinated ethylene propylene (FEP) films for curing. For the study of light-responsive flexible devices, the transformation of the hydrogel spatial shape can be achieved by first printing a planar structure through the light-curing platform shown in the figure. The FEP film is pasted on two coverslips to maintain the shape. The thickness of curing is determined by the coverslips between the FEP films.

The PNIPAM hydrogel prepolymer solution is rapidly formed on the curing platform according to the designed photomask pattern. The CNTs are added by dip-dyeing, which is similar to the form of dyeing cloth or dyeing ink on rice paper in Chinese calligraphy. The process of adding CNTs is shown in Figure 1C. First, the cured hydrogel structure is put into hot water at 40 °C. Overall, the hydrogel will exhibit hydrophobicity, which will drain water and shrink in volume. Then, it is put into the solution of CNTs dispersed by water. The temperature is 15 °C, which is lower than LCST. The hydrogel will again exhibit hydrophilicity overall, thus absorbing water and expanding in volume. In the process of water absorption, CNTs are then brought into the interior of the hydrogel. The hydrogel impregnated with carbon nanotubes will shrink and swell alternately in hot and cold water many times, and the poorly bound CNTs can be removed this way. The composite hydrogel did not shed CNTs during frequent use. One of the reasons may be due to the tendency of CNTs to have certain unidirectional transport after penetrating into the hydrogel. When the hydrogel absorbs water in cold water, the hydrogel swells and the pores become larger, making it easy for the movement of CNTs to the interior. In hot water, or after local exposure to light, the hydrogel shrinks and the pores decrease, which will inhibit the process of CNT outflow. The photo-responsive hydrogel after two months of placement can still perform well in photo-response. The PNIPAM hydrogel can have photo-responsive properties after the CNTs are added using the above method.

### 2.2. Study of the Deformation Properties of PNIPAM/CNT Hydrogels

**Figure 2:**
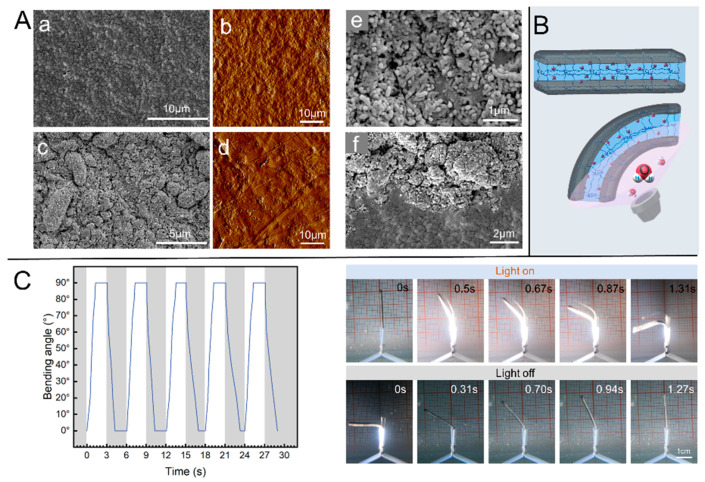
(**A**) Characterization of the hydrogel structure. (**a**,**c**) SEM photographs without and with CNTs, respectively, and (**b**,**d**) AFM photographs without and with CNTs, respectively. (**e**) A high-resolution SEM photograph of the surface of the hydrogel with CNTs. (**f**) A side SEM photograph of the hydrogel. (**B**) Schematic diagram of the light-driven deformation principle of composite hydrogel. (**C**) Light-controlled bending deformation performance.

The successful incorporation of CNTs is essential for the hydrogel to have photo-responsive properties. Since the CNTs are added by late dipping, the particle size of the CNTs will have an important effect on the effect of their attachment inside the hydrogel. The surface of the hydrogel impregnated with multiwalled carbon nanotubes (MWCNTs) showed a darker color and better photo-thermal conversion than that of the hydrogel impregnated with single-walled carbon nanotubes (SWCNTs). The SEM and AFM photographs of the hydrogels with and without the addition of MWCNTs are shown in Figure 2A. It can be seen from the SEM image of the hydrogel after drying (Figure 2A(c)) that the MWCNTs are agglomerated on the surface of the hydrogel. Through the analysis, we conjecture that MWCNTs, due to the large particle diameter and long length of the individual chain structure of CNTs, only a part of one end penetrates deep into the hydrogel, and the rest will remain outside, thus showing the agglomerated state after drying. This will make the surface of the hydrogel exhibit a darker color. A clear contrast can also be seen in the microscope photographs of Figure 2A with and without the addition of CNTs. As Figure 2A(e,f) shows the high-resolution SEM photographs of the surface and side of the hydrogel, the state of CNTs aggregated on the surface can be clearly seen in Figure 2A(e), and the exposed end of the CNTs is clearly visible. The CNTs show an intertwined and entangled state, agglomerated on the surface of the hydrogel. The demarcation line between the CNTs and hydrogel can be seen in Figure 2A(f). One end of the bundle of CNTs can be faintly seen wrapped inside the hydrogel near the demarcation line. The CNTs are not easily dislodged during the repeated washing and deformation cycles, which indicates that the CNTs are firmly attached. The dense end surfaces of CNTs can be seen on the surface of the hydrogel, and there must be one end that is deep inside the hydrogel. The principle of light-driven deformation of the PNIPAM/CNT composite hydrogel is shown in Figure 2B. One side of the hydrogel is irradiated by a xenon light source, and since the composite hydrogel is impervious to light, the backlit side receives no energy from the light source. On the light irradiated side, the CNTs are instantaneously heated up by the photothermal effect, and the temperature of the backlight side does not change for a short time. This results in a temperature gradient on both sides of the hydrogel, and the increase in temperature on the side irradiated by the light source, which easily exceeds the LCST of PNIPAM, will cause the hydrogel on this side to discharge water and shrink in volume. There is no change on the other side. When the light is removed, the surrounding water causes the locally heated surface to dissipate heat and the temperature falls below the LCST, and the hydrogel changes back to the water-absorbing state and the shape is restored. The photo-responsiveness of the PNIPAM/CNT composite hydrogel is verified by printing the hydrogel rectangular structure. As shown in Figure 2C, the vertically placed hydrogel rectangular structure can maintain an upright state when there is no light irradiation, indicating that the hydrogel has a good ability to retain its shape in water. When one side of the hydrogel was irradiated with a xenon light source with a light intensity of about 20 kW/m^2^, the light-responsive hydrogel could bend to a state larger than 90° in as early as about 1.3 s. Withdrawing the light, the photo-responsive hydrogel can return to its initial state in about 1.27 s. The process of frequent bending and deformation of the hydrogel was controlled by light. After conducting the experiment for dozens of cycles, the deformation property of the hydrogel was basically unchanged. By measuring the time for the deformation to reach each angle, we intercepted five cycles of the process for data collation and came up with the data graph shown in Figure 2C. From the change in the slope of the curve in the graph, it can be seen that the deformation in the initial stage is faster in both the bending and recovery processes. The difference in time between the bending deformation and the recovery phase is not significant. The light-controlled bending process is influenced by the energy of light. When there is no light, the speed of the recovery process of the hydrogel depends entirely on the manufactured hydrogel itself. In the experiment, it can be seen that the speed of its recovery is very fast. Applying it in the fishtail, the recovery ability of the light-controlled flexible drive unit and the light response ability are both important for the oscillation of the fishtail (Video S1, Supplementary Material).

**Figure 3:**
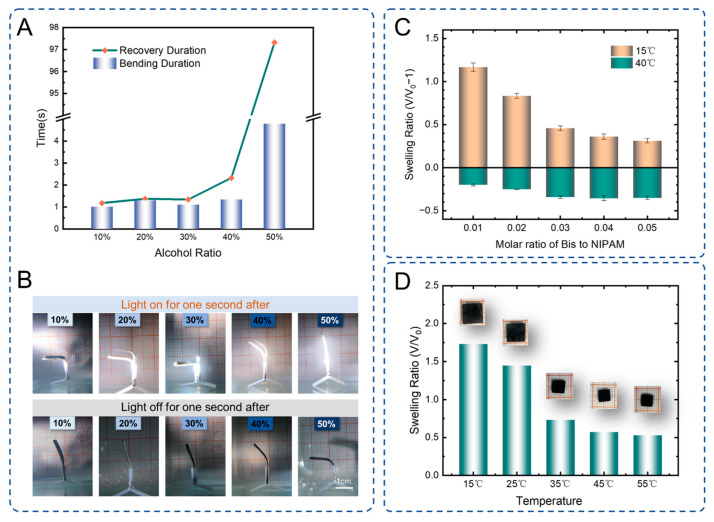
(**A**) The effect of different concentrations of alcohol as solute on the deformation properties of hydrogels. (**B**) Comparison of bending deformation and recovery states of hydrogel samples with different concentrations of alcohol as solute after one second of light exposure and one second of light off. (**C**) The effect of molar ratio of crosslinker to monomer on the swelling rate of hydrogels. (**D**) Effect of the concentration of NIPAM on the swelling rate of hydrogels.

In the course of the experiments, we found that the solvent in the form of miscible water and ethanol performed better than a single material as a solvent. A comparison of the bending time and recovery time of PNIPAM hydrogels with different concentrations of alcohol as a solvent is shown in Figure 3A. The light response is faster for all alcohol concentrations between 10% and 40%. After the concentration of 40%, the response of the hydrogel becomes significantly slower. For 10% and smaller percentages of alcohol solution, the chemicals will not dissolve sufficiently. Therefore, we chose the percentage of alcohol solvent to be in the approximate range of 20% to 30%. The process of the study also characterized the performance of the hydrogel by the speed of bending and recovery of the hydrogel strips. Making the hydrogel irradiated for one second and recovered for one second, the effect of different concentrations of alcohol on the deformation properties of the hydrogel can be clearly seen in Figure 3B. The solutes in the prepolymer solution are including three parts, NIPAM, N,N′-methylenebis(acrylamide) (Bis) and water-soluble diphenyl(2,4,6-trimethylbenzoyl)phosphine oxide (TPO). The process of their dissolution in the solution is due to the molecular forces with the solvent molecules. The dispersion state of ethanol molecules and water molecules in the solute, or the agglomeration effect of water molecules, also affects the distribution of the chemicals. In the process of light curing, the polymer chain growth is very fast. The distribution of chemicals also affects the interwoven state of the chain structure. This in turn affects the process of temperature-responsive water transport within the PNIPAN hydrogel. 

During the formation of long chains of the NIPAM monomer, the cross-linking agent Bis can join the two chains together by grafting reaction. The state of the polymer chain can have a pretty big impact on the responsive performance of PNIPAM hydrogels. Therefore, the application of PNIPAM to flexible actuator parts requires finding the right ratio of BIS to NIPAM. Five sets of control experiments were set up by controlling the concentration of NIPAM monomer constant and changing the content of Bis. When the hydrogels were put into cool water at 15 °C and hot water at 40 °C, respectively, the change in the swelling rate can visualize the looseness of the hydrogels. It can be seen from Figure 3C that the rate of change in the volume of the hydrogel caused by the temperature will be smaller as the cross-linking agent increases. The sum of the two-color bar graphs shown indicates the maximum deformation range of the hydrogel. Flexible actuators require a large amount of deformation. The molar ratio of BIS to NIPAM is 0.01, but the structure formed at this time is too soft to keep its shape well because there is too little cross-linker. The molar ratio of Bis to NIPAM was chosen around 0.02. The temperature response characteristics of the final configured hydrogel are shown in Figure 3D. The comparison of the dimensions of the same square hydrogel at different temperatures clearly shows the change in volume exhibited by the hydrogel as the temperature changes. The produced hydrogel has a better deformation ability. The transition is maximum between 25 °C and 35 °C, as 32 °C is the LCST of PNIPAM hydrogel, which is in general agreement with the properties of other studies on PNIPAM hydrogels. The deformation rates of hydrogels at different temperatures are affected by the structure and initial state, and the deformation rates presented at different temperatures are not easy to measure. The difference in temperature during deformation, as well as the interval in which the deformation is switched, can make the deformation rate different. When increasing and decreasing the LCST temperature, the hydrogel shows a large deformation due to the overall water absorption presented as well as the hydrophobic properties. Therefore, in our experiments, the main focus is on the deformation rate when increasing and decreasing the LCTS temperature. In order to achieve faster deformation rate and larger deformation of the hydrogel, we conducted a large number of experiments during the exploration of the ratio of chemicals. One of the main verifications is the speed of water absorption recovery when switching from the environment of hot water at about 50 °C to water at about 25 °C. During the search for the best formulation, the hydrogel generally drained and contracted quite fast in hot water. Most of the failed formulations had a more desirable shrinkage speed in hot water but a particularly slow recovery speed when transferred to a room temperature environment in water. We eventually arrived at a more ideal formulation of the hydrogel prepolymer solution. The recovery time of the hydrogel for water absorption in room temperature environment reached about 1.3 s (Figure 2C).

### 2.3. Fabrication of Bionic Micromanipulator

**Figure 4:**
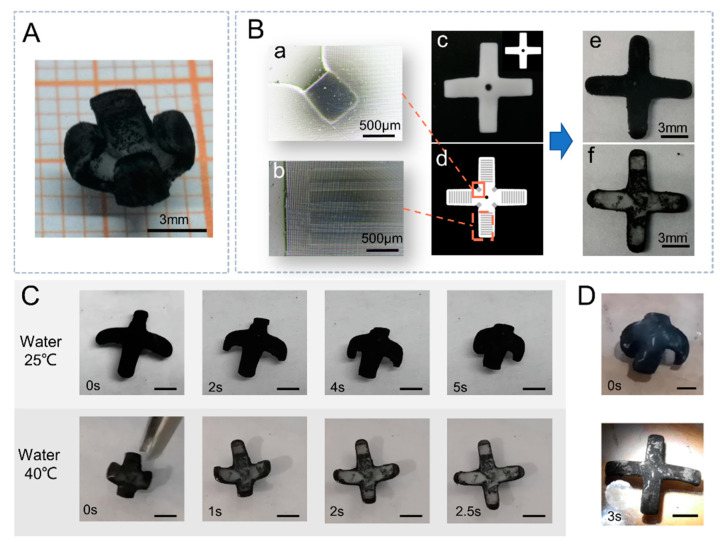
(**A**) Normally closed micromanipulator. (**B**) Multilayer structure of the micromanipulator. (**c**) The deformation layer of the manipulator with the photomask pattern of the fabrication process in the upper right corner. (**d**) The photomask pattern of the rigid layer with the arrangement of gray values to guide the direction of deformation. (**a**,**b**) Photographs of the optical microscope of the rigid layer. (**e**) Photograph of the deformation layer side of the bilayer structure of the micromanipulator. (**f**) Photograph of the rigid layer side of the micromanipulator. (**C**) Deformation behavior of the micromanipulator driven by temperature (Scale bar is 3 mm). (**D**) Deformation of the light-driven micromanipulator (Scale bar is 3 mm).

Drawing on the principle of 4D printing, a light-driven micromanipulator, as shown in Figure 4A, was fabricated from the PNIPAM/CNT composite hydrogel. For the fabrication of the normally closed micromanipulator, we used a two-layer structure, as shown in Figure 4B. The light-responsive deformation layer is made by the PNIPAM/CNT composite hydrogel with good deformation performance (Figure 4B(c)). The material of the rigid layer is also PNIPAM; however, the material used is a dense structure that does not undergo volume changes during temperature or light exposure. The two layers are structured with the same type of material, and the different states of interweaving of the internal polymer chains lead to differences in physical properties. Figure 4B(e) shows one side of the deformation layer, where CNTs are successfully added. Figure 4B(f) shows the side of the rigid layer, where only a small amount of CNTs are attached. The photomask pattern of the rigid layer is shown in Figure 4B(d), and different positions have different gray values. During the light-curing process, the photomask patterns with different gray values for the same light time result in different crosslinking of the hydrogel. Darker areas also correspond to less light intensity. The rigid layer is set at a smaller gray value of 140 at the location of the bent and deformed joints as shown in the figure. The rest is 255. The demarcation line between the structures with different gray values can be clearly seen in Figure 4B(a,b). The sequence of light curing caused by different gray values can also be different. The brighter regions, where the photo-initiator is the first to collect full energy, initiate the polymerization reaction of the polymer. Darker areas cure later. The Young’s modulus of the hydrogel formed by curing in the brighter areas is also higher than that in the darker areas for the same curing time. The darker areas, shown in Figure 4B(d), are more likely to deform under the action of external forces. This can serve as a guide for the subsequent deformation through the reasonable arrangement of the gray value pattern. As shown in Figure 4C,D, the temperature response process and light response process of the micromanipulator are shown, respectively. The micromanipulator can complete the light-driven or temperature-driven opening action in about 3 s and the closing action in room temperature in 5 s. The slow recovery of the micromanipulator shown in Figure 4C at about 25 °C is due to the constraint of the rigid layer. The initial fabrication state of the rigid layer is a planar structure. When the manipulator bends at room temperature, the deformation layer also needs to drive the rigid layer to bend. Frequent stimulus responses can be performed to complete the opening and closing actions. The light-driven, normally closed micromanipulator has great potential for flexible grasping tasks of tiny objects (Video S2, Supplementary Material).

**Figure 5:**
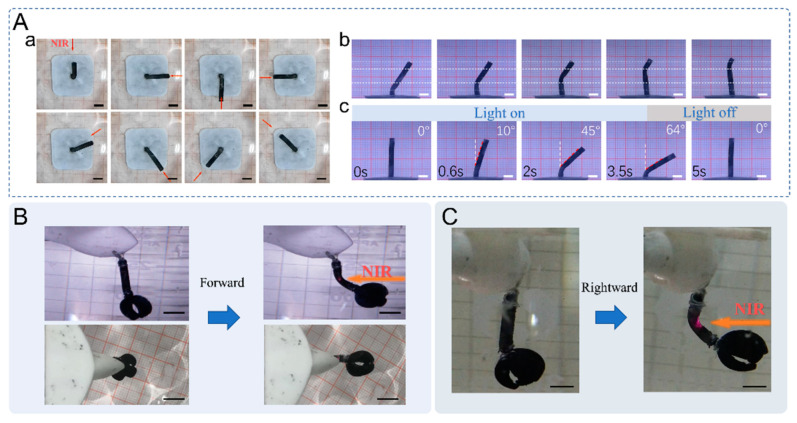
(**A**) NIR laser t-driven performance of the multi-degree-of-freedom arm. (**a**) The NIR laser drives the bionic arm to rotate 360°. (**b**) The NIR laser can drive the bionic arm to deform at any position. (**c**) The effect of light duration on the amount of bending deformation of the bionic arm. (**B**) The bionic arm drives the micromanipulator to achieve a forward bending motion. (**C**) The bionic arm drives the micromanipulator to achieve a rightward bending motion (Scale bar is 5 mm).

With the good heat production efficiency of the NIR laser with CNTs and the ability of the NIR laser to localize irradiation, we fabricated a multi-degree-of-freedom bionic arm as shown in Figure 5. The free end of the bionic arm can reach an arbitrary position close to a hemispherical surface. As shown in Figure 5A, changing the direction of NIR irradiation, the bionic arm can rotate 360° in the horizontal plane and turn to an arbitrary direction (Video S3, Supplementary Material). The area of NIR irradiation is a point, and the photothermal conversion process also occurs at the location of the NIR spot. After the light is turned off, the bionic arm can quickly return to its pre-deformation state because the spot size at the irradiated location is small and can be quickly cooled by the surrounding water. By irradiating different positions of the bionic arm, any position can be used as a joint for rotation. This fully utilizes the advantages of the PNIPAM/CNT photo-responsive hydrogel as a flexible actuator. As shown in Figure 5A(c), the adjustment of the bending angle can also be achieved by controlling the length of irradiation time. The longer the irradiation time, the larger the bending angle of the bionic arm. After the NIR light source is turned off, the bionic arm can quickly return to the straightened state in about 1.5 s. One end of the bionic arm is fixed in front of the swimming soft robot, and the other end is connected to the micromanipulator. The bionic swimming soft robot can then control the swing of the arm via NIR to adjust the position and posture of the micromanipulator (Video S4, Supplementary Material). The process of swinging the arm to the front and swinging the arm to the right of the soft robot is shown in Figure 5B,C, respectively. The light-controlled bending of the bionic arm can drive the micromanipulator to an arbitrary position in a hemisphere surface. The difference in the light intensity and irradiation position can further expand the flexibility of the micromanipulator.

### 2.4. Various Application Scenarios of Bionic Soft Robot

**Figure 6:**
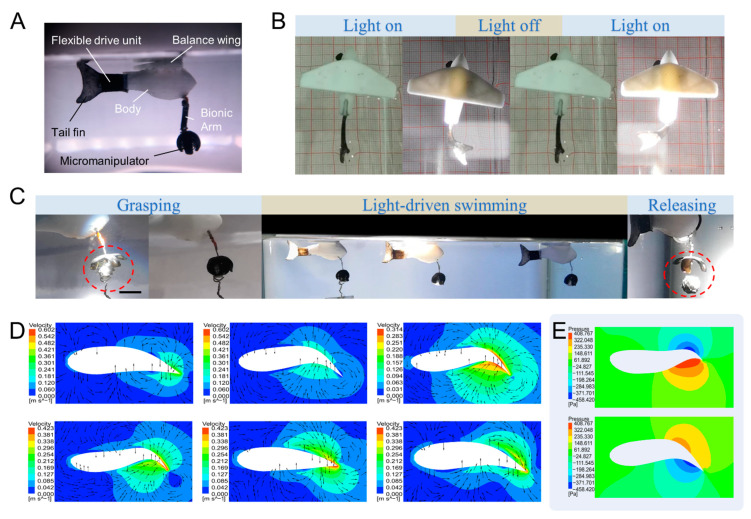
(**A**) The main components of the light-driven microfish. (**B**) Alternating left and right illumination of the light source to realize the attitude adjustment of the fish tail swaying from left to right. (**C**) The light-driven microfish realizes the task of carrying objects from the starting point to the end point. The light-driven grasping of the goods is realized at the starting point, the light-driven the microfish swim, and then the light-driven release of the goods is realized at the end point. (**D**) Finite element analysis of velocity field of fish swimming. (**E**) Pressure field analysis of fish swimming (Scale bar is 5 mm).

Using PNIAPM/CNT photo-responsive hydrogel materials as photo-responsive actuators, we fabricated a light-drivable bionic swimming soft robot as shown in Figure 6A. The bionic swimming soft robot mainly consists of a flexible drive unit at the tail part made of light-responsive hydrogel, a tail fin, a fish body, a balance wing, a bionic arm and a bionic micromanipulator. The tail fin and the flexible drive unit are integrally formed by the light-curing equipment shown in Figure 1. This way, when the xenon light shines on the tail of the fish, only the position of the flexible drive unit bends, which then drives the tail to swing and paddle the water. The fish body and the balance wing are made by fused deposition molding (FDM) 3D printing. The application of balance wings draws on the principles of fixed-wing aircraft flight. It can restrain the freedom of the fish body to roll sideways. The fish tail was designed to keep vertical to make the attitude of the fish body more stable. The light can easily irradiate the two sides of the fish tail, which is convenient for the control of the fish tail drive. The addition of the bionic micromanipulator can realize the task of carrying objects during the movement. The main forms of movement of fish in nature are “S”- and “C”-shaped swimming. In the process of making and controlling the swimming of the bionic swimming soft robot, we have borrowed the principle of “C”-shaped swimming. Figure 6B shows the control process of the left–right oscillation of the fish tail controlled by the surface light source. The fishtail bends to the side of the light and recovers after the light source is turned off. Two xenon light sources of optical fibers alternately irradiate the two sides of the fish tail to realize the left–right oscillation of the fish tail. The photo-responsive hydrogel of the flexible drive unit drains and shrinks in volume upon exposure to light. After turning off the light source, the hydrogel will again absorb water and expand in volume. This process is equivalent to the muscles on both sides of the fish’s tail, alternately contracting and stretching. The process of the bionic microfish performing the object handling task is shown in Figure 6C (Video S5, Supplementary Material). First, the micromanipulator is opened by shining a light source at the position of the normally closed micromanipulator. The open micromanipulator is positioned above the object, the light source is turned off, and then the micromanipulator closes to achieve object grasping. Next, after reaching the end point, the light source is used to illuminate the micromanipulator once again to achieve the release of the object. All positions are driven by light illumination, and the controllability of the light source irradiation position makes each driving action non-interfering with each other. It is even possible to achieve joint control of light-driven deformation behavior in multiple positions. This is not possible with the entire range of stimulation forms such as magnetic drive.

Through the finite element analysis, we can clearly see the state of the flow field where the fish is located when it swims (Video S6, Supplementary Material). Figure 6D shows the analysis of the velocity field of the fluid and the state of the flow field when the fish reaches a specific attitude during the tail swing and recovery, respectively. The red area of the diagram represents the fluid with the highest velocity. Further analysis shows that the overall direction of motion of the flow field in the red region is rearward. In other words, the fluid is plucked to move backward at high speed by the swing of the fish tail. The fish tail out discharges the backward momentum, and the fish can gain forward momentum. Figure 6E shows the pressure field analysis and the posture of the high-pressure flow field acting on the local area behind the fish tail, in terms of force can also be seen microfish can obtain forward momentum. The designed streamlined fish body and the trailing angle at the position of the leading edge of the balanced wing can reduce the drag of the fluid when the microfish is moving forward.

## 3. Materials and Methods

### 3.1. Materials

N-Isopropylacrylamide (NIPAM, 98%, with stabilizer MEHQ) and N,N′-methylenebis (acrylamide) (Bis, 99% purity) were purchased from Shanghai Aladdin Biochemical Technology Co., Shanghai, China. Water-soluble TPO-based nanoparticle photoinitiators (photoinitiators) were purchased from Sigma-Aldrich, Oakville, ON, Canada. The water-dispersible photoinitiator nanoparticle contains 10% (*w*/*w*) of the type I photoinitiator diphenyl(2,4,6-trimethylbenzoyl)phosphine oxide (TPO). CNTs (MWCNTs, purity: >95 wt%, inner diameter: 3–5 nm, outer diameter 8–15 nm, length 3–12 μm) were purchased from Suzhou Tanfeng Graphene Technology Co., Suzhou, China.

### 3.2. Preparation of PNIPAM Hydrogel Prepolymer Solution

Configure a prepolymer solution for the flexible-driven PNIPAM hydrogel. In total, 300 μL of anhydrous ethanol was mixed with 700 μL of deionized water as the miscible solvent and ultrasonically dispersed in an ice bath for ten minutes. Overall, 480 mg of NIPAM, 13 mg of Bis and 12 mg of TPO were placed in the miscible solvent and dispersed by magnetic stirrer for 15 min, followed by ultrasonic dispersion in an ice bath for two minutes. A total of 8 mg of CNTs and 0.8 mg of dispersant were sonicated and dispersed in 1 mL of deionized water for 20 min. The solvent of the PNIPAM prepolymer solution for the hydrogel rigid layer was 850 μL of anhydrous ethanol mixed with 150 μL of deionized water, and the solutes were 800 mg NIPAM, 30 mg Bis and 12 mg TPO. Dispersion was carried out the same way as described above. 

### 3.3. Fabrication of Light-Driven Microfish

Design the photomask pattern for the fish tail and the micromanipulator, the configured prepolymer solution was cured in the light-curing device shown in Figure 1 for 10 s. The area of the micromanipulator with a grayscale value of 140 was added by Photoshop software. The prepolymer solution was placed into a silicone catheter with an outer diameter of 2 mm and an inner diameter of 1.5 mm for curing to create the bionic arm. Before adding the prepolymer solution, the side of the silicone catheter was cut to facilitate the removal of the cured hydrogel structure. Because of the hydrophobicity and shape retention ability of the silicone catheter, the sides will be tightly bound even after the cut, and no prepolymer solution will flow out. The cured hydrogel structure is added with CNTs by dipping, as shown in Figure 1C. After adding CNTs, the hydrogel structure is then expanded and contracted several times in clean hot and cold water alternately to remove the poorly attached CNTs. The microfish body is created using 3D printing in FDM form. The fish tail, the bionic arm and the bionic micromanipulator are assembled into the microfish by designing the structure in the form of insertion and bonding.

### 3.4. Surface Light Source and Point Light Source Coupling Drive

The surface light source is provided by a xenon lamp light source, which is purchased from Bebei Lighting. The point light source is provided with the help of a near-infrared near-light source, and the NIR laser is purchased from Fuzhe Technology Co. Changsha, China. The wavelength is 808 nm and the power is 500 mw. The xenon light source leads to an optical fiber, which irradiates the fish tail and controls the swing of the fish tail. The NIR laser precisely controls the oscillation of the arm.

### 3.5. Finite Element Analysis of Microfish Swimming

Firstly, the 2D models of the streamlined fish and the light-driven microfish are simplified, respectively. After importing the models in the finite element simulation software Fluent, the model is meshed and the boundary conditions are set. Custom functions are set to realize the oscillation of the fish tail by moving the mesh. Finally, the analysis is calculated and the velocity and pressure clouds are plotted.

## 4. Conclusions

In this study, we present a novel light-driven swimming soft robot with multiple functions. A simple method is proposed to make the temperature-sensitive hydrogel PNIPAM with photo-responsive properties and hydrogel structures are fabricated using a DMD-based light-curing system. This project makes full use of the rapid prototyping capability of complex structures by light-curing manufacturing technology. Many synthetic methods on photo-responsive hydrogels combine heat-converting materials with temperature-sensitive hydrogels. However, they generally mix a material with good photo-thermal conversion such as a carbon-based material with a hydrogel prepolymer solution at the same time, and then mold it through a mold. This paper is dedicated to exploit the molding ability of light curing to fabricate photo-responsive hydrogel structures. CNTs can affect the molding ability of light curing. In this paper, we first perform the fabrication of complex structures of temperature-sensitive hydrogels by a light-curing 3D printing technique. Then, CNTs are firmly fixed on the hydrogel surface by leveraging the characteristics of water absorption and drainage of PNIPAM hydrogels, and also by combining the small diameter and long size of carbon nanotubes. This approach has the advantage of fabricating complex structures of photo-responsive hydrogels. The formulation of a prepolymer solution suitable for light-curing fabrication with a fast response was found through extensive comparative experiments. By summarizing the failed configuration schemes, a PNIPAM hydrogel configuration suitable for fabricating rigid layers was derived. The rigid layer fabrication scheme turns the disadvantage of formulations of PNIPAM hydrogels that cannot respond to stimuli into an advantage. The photo-responsive layer was fabricated by PNIPAM hydrogels with good deformation properties. The same material is used for both, only the ratio is different. The same type of material has good compatibility and the rigid layer is more firmly attached to the photo-responsive layer. The fishtail and the micromanipulator are made by bonding the rigid layer and the light-responsive layer. The rigid layer material also has good light-curing molding ability and is also a good bonding agent between the two structures. The reciprocal oscillation of the fish tail can be well driven by the xenon light source to realize the swimming of the soft robot which is also verified by simulation analysis. In addition to the swimming motion, the bionic swimming soft robot can also realize the oscillation of the bionic arm driven by the coupling of point and surface light sources and the object grasping motion of the micromanipulator. The normally closed micromanipulator can carry objects during the swimming of the microfish to realize the transportation task. This project is designed with a rational structure to better achieve the multitasking capability of the soft robot. The soft robot is capable of both locomotion and object transfer. The arm can also be swung in any direction. Xenon lamp and NIR multisource coupling drive can realize simultaneous drive for motion and task execution. The flexibility of local drive in the form of light as a drive is fully exploited. This work provides new ideas for the development of underwater microrobots and flexible bionic robots. The photo-responsive PNIPAM/CNT composite hydrogel has great prospects for application.

## Data Availability

The datasets generated during and analyzed during the current study are available from the corresponding author on reasonable request.

## Supplementary Materials

The following supporting information can be downloaded at: https://www.mdpi.com/article/10.3390/ijms23179609/s1.
